# Intrapartum PCR assay versus antepartum culture for assessment of vaginal carriage of group B streptococci in a Danish cohort at birth

**DOI:** 10.1371/journal.pone.0180262

**Published:** 2017-07-05

**Authors:** Mohammed Rohi Khalil, Niels Uldbjerg, Poul Bak Thorsen, Jens Kjølseth Møller

**Affiliations:** 1 Department of Gynecology and Obstetrics, Lillebaelt Hospital, Kolding, Denmark; 2 Department of Obstetrics and Gynecology, Aarhus University hospital, Skejby, Denmark; 3 Research Unit for Gynecology and Obstetrics, Department of Clinical Research, University of Southern Denmark, Odense, Denmark; 4 Department of Clinical Microbiology, Lillebaelt Hospital, Vejle, Denmark; Universidade de Lisboa Faculdade de Medicina, PORTUGAL

## Abstract

The aim of this study was to compare the performances of two strategies for predicting intrapartum vaginal carriage of group B streptococci (GBS). One strategy was based on an antepartum culture and the other on an intrapartum polymerase chain reaction (PCR). We conducted a prospective observational study enrolling 902 pregnant women offered GBS screening before delivery by two strategies. The Culture-strategy was based on vaginal and rectal cultures at 35–37 weeks’ gestation, whereas the PCR-strategy was based on PCR assay on intrapartum vaginal swab samples. An intrapartum vaginal culture for GBS was used as the reference standard from which the performances of the 2 strategies were evaluated. The reference standard showed a GBS-prevalence of 12%. The culture-strategy performed with a sensitivity of 82%, specificity of 91%, positive predictive value (PPV) of 55%, negative predictive value (NPV) of 98%, and Likelihood ratio (LH+) of 9.2. The PCR-strategy showed corresponding values as sensitivity of 83%, specificity of 97%, PPV of 78%, NPV of 98%, and LH+ of 27.5. We conclude that in a Danish population with a low rate of early-onset neonatal infection with GBS, the intrapartum PCR assay performs better than the antepartum culture for identification of GBS vaginal carriers during labor.

## Introduction

Even though early-onset neonatal infection with Group B streptococci (EOGBS) is rare, it still constitutes a health problem in countries where the prevalence of EOGBS disease is 2 in 1,000 live births, and the mortality rate is 50% [1]. As EOGBS [2] occurs only among the group of neonates who are born by the 10–35% of women colonized vaginally with GBS [3–8], the Centers for Disease Control and Prevention (CDC), USA, in 2002 recommended universal culture screening of all pregnant women between 35 and 37 weeks’ gestation in order to give intrapartum antibiotics to the screen positives [9, 10]. The implementation of this strategy was followed by a decrease in the EOGBS rate from 1.5 to 0.4/1,000 live births [9]. This decrease must be categorized as a success, however, one might wonder why the EOGBS rate in some other countries including Denmark is only 0.1–0.4/1,000 live births [11] even though they have not implemented this antepartum culture-based screening program.

A weakness of the CDC strategy based on a rectovaginal culture obtained often weeks before labor is that shifts in the GBS status [2] reduces the sensitivity to about 50% [12] and positive predictive value to about 60% [13, 14]. This explains why the majority of neonates with EOGBS in the USA are born by women with a negative test for GBS [15–17]. Furthermore, it may cause an overuse of antibiotics if a test for GBS has been positive at a preterm screening but GBS is no longer present at delivery [18, 19]. However, changes in the GBS colonization status of the mother during the period between antepartum screening and delivery may be influenced by several factors including a low colonization status of the woman, suboptimal timing of specimen collection, and inappropriate transport media for specimens, such as lack of storage at 5°C if transportation of specimens for culture is delayed or there is inadequate laboratory processing [20].

These data call for a rapid GBS test that can be used intrapartum for better identification of women carrying GBS in the vagina at the time of delivery. Previous studies have shown that the sensitivity of the intrapartum polymerase chain reaction test (PCR) to detect GBS colonization during labor may be superior to antenatal cultures; however, these differences have not always been statistically significant [14, 21–23].

The BD Max GBS assay (BD Diagnostic Systems, Québec, Canada) performed on the BDMax^™^ system (BD Diagnostic Systems, Sparks, MD) is a PCR test intended for use with enriched Lim broth culture after 18 h of incubation of vaginal/rectal swab samples and can provide results for up to 24 concurrent specimens in approximately 2.5 h. The use of E-Swab samples with the BDMax^™^ GBS assay, eliminating the Lim broth inoculation and incubation steps, may enable a rapid detection of GBS in pregnant women at birth with a sensitivity of 93% [24]. The performance of such an intrapartum PCR-test without a prior enriched Lim broth culture, however, has never been evaluated in a Danish population of pregnant women with a low risk of their babies acquiring early-onset neonatal group B streptococcal disease.

Therefore, the aim of this study was to compare the performance of an antepartum culture based screening strategy and an intrapartum PCR assay for the prediction of intrapartum vaginal carriage of group B streptococci (GBS) in a Danish cohort, using intrapartum vaginal culture as the reference standard.

## Material and methods

**Fig 1:**
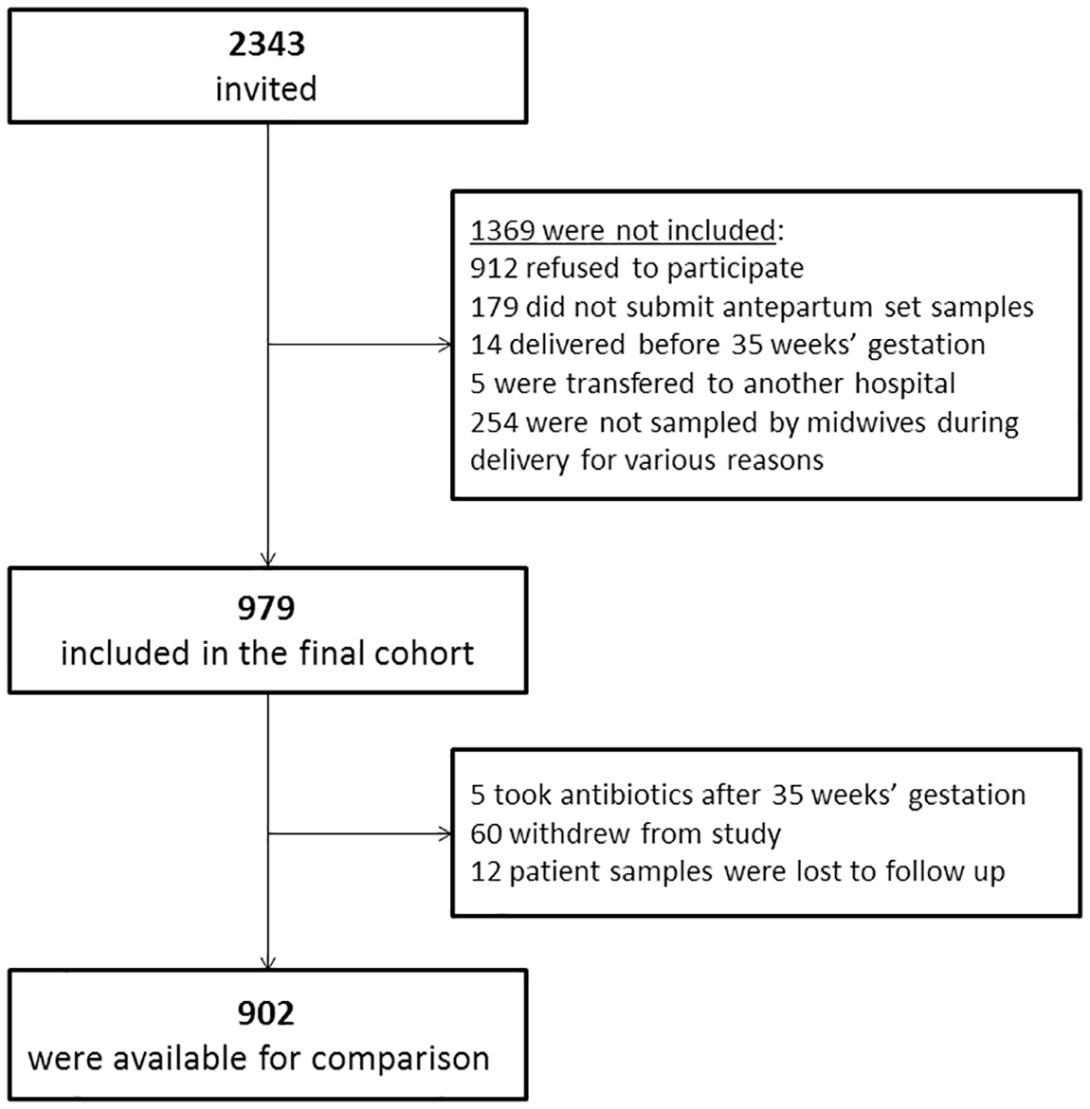
Flowchart of the population.

A total of 2,343 pregnant women attending the prenatal clinic at Lillebaelt Hospital, Kolding, Denmark (with an average of 3,200 deliveries per year) over a 15 months’ period between April 2013 and June 2014 were invited to participate in this prospective observational study. One thousand three hundred sixty-four (n = 1,364) declined to participate, leaving 979 participants in the final cohort (Fig 1). Detailed information on oral antibiotic use during pregnancy was obtained from registered data in both patient hospital records and the Danish Medical Agency’s Register of non-hospitalized patient use, which included records on all drug prescriptions filed at any Danish pharmacy. Five patients received antibiotics after week 35 of gestation and were therefore excluded (Fig 1). Further, sixty women withdrew from the study at the time of birth for various reasons. Twelve were lost for follow-up. Thus, 902 sets of patient samples were available for comparisons between antepartum culture (culture-based screening) and PCR analysis (PCR-based screening) with intrapartum culture.

### Inclusions criteria

All pregnant women attending the prenatal Clinic at Lillebaelt Hospital, Kolding were invited to participate.

### Exclusions criteria

Women who delivered preterm (< 37 weeks´ gestation)

Women who received antibiotics after 35 weeks´ gestation

Women with communication restrictions

Women under 18 years old were excluded.

### Collection of specimens

All samples were collected using nylon flocked swabs submerged separately into 1 ml of E-Swab transport medium (E-Swab, Copan Diagnostics, Brescia, Italy).

- The Culture-strategy: At 35–37 weeks’ gestation, each participant obtained a self-administered and time-saving vaginal and rectal swab sample for culture during a planned visit to the outpatient clinic [25, 26].
- The reference standard: During labor, the midwife collected a vaginal swab sample which was used for immediately culture of GBS.
- The PCR-strategy: The vaginal swab obtained during labor as intrapartum culture sample (reference standard), submerged into the transport medium, was frozen at minus 80°C for later GBS PCR analysis as a batch processing.

In addition to the written information with text and drawings on how women should obtain a self-administered vaginal and rectal swab sample for the culture, two instructional videos were available to all participants on the project website. The sampling was carried out by inserting and rotating one E-Swab 1.5–2 cm inside the vagina and another one in the rectum by inserting the swab 1.5–2 cm beyond the anal sphincter. All samples were analyzed at the Department of Clinical Microbiology, Lillebaelt Hospital, Vejle, Denmark.

### Culture of specimens

Samples were cultured at the time of arrival to the laboratory; if received after 8 PM, they were kept at 4°C until the next morning. Broth enrichment was not employed as part of a strategy to simulate and evaluate a rapid testing of the presence of GBS in the vaginal samples by both culture and PCR. Therefore, direct plating without prior enrichment of the specimen in a culture broth was carried out by streaking the E-Swab specimen on a selective Granada agar plate. The vaginal and rectal swabs from the same patient were seeded on different sides of the same Granada agar plate (BioMérieux^®^, Spain). The Granada agar plates were incubated immediately after seeding in the CO_2_-containing atmosphere at 35°C. The specimen tubes containing the vaginal intrapartum E-Swab sample medium were subsequently frozen at minus 80°C for later PCR analysis. The Granada agar plates were read after one and two days of incubation.

All GBS-like colonies (identified by their orange color on Granada agar plates) were routinely confirmed as *Streptococcus agalactiae* using the Microflex LT ^™^ MALDI-TOF system (Bruker Daltonik, Germany). A semi-quantitative culture assessment of GBS growth was conducted in most cases. The culture was classified as having only growth of few GBS colonies (+), some (++) or many (+++) by intrapartum vaginal culture.

### Polymerase chain reaction (PCR)

PCR analysis is a real-time PCR test performed on the BDMaxTM system (Becton, Dickinson and Company, USA) without enrichment of the specimen in a culture broth prior to analysis. The BDMax^™^ System automatically extracts the target nucleic acid and amplifies a section of the cfb gene sequence of the GBS genome if present. The BDMax^™^ Assay includes an Internal Process Control to monitor for the presence of potential inhibitory substances as well as system or reagent failures that may be encountered during the entire process. The results are reported by the BDMax^™^ software as a qualitative answer, either positive or negative for GBS. In a small number of cases, the specimens were initially undetermined because of inhibition, reagent failure or system errors, which led to additional testing by taking a new aliquot of the sample and repeating the DNA extraction and PCR assay. The PCR analyses were performed retrospectively on frozen samples as batch processing.

The results of the GBS culture and PCR tests were read by independent laboratory technicians and recorded separately.

### Formalities

The study was approved by the Regional Scientific Ethical Committees for Southern Denmark (S-20130089) and the Danish Data Protection Agency (2008-58-0035). The date of issue: 6 November 2013. All participants provided written informed consent.

### Statistics

STATA Statistics/Data Analysis software (version 14; StataCorp LP) was used for the statistical analysis. Sensitivity, specificity, positive predictive values (PPV), negative predictive values (NPV), and Likelihood ratio (LH) including 95% confidence intervals (CI) were calculated for both antepartum GBS screening and the intrapartum PCR assay using culture of a vaginal swab sample as the gold standard.

## Results

All 979 enrolled women had an antepartum swab obtained as part of the culture-strategy and 902 (92%) had an intrapartum swab as part of the culture-based reference standard and the PCR-strategy. The intrapartum vaginal GBS colonization rate detected by culture was 11.5% (reference standard). By comparison, the culture-based strategy found 9.4% (85/902) GBS-positive women by combining results from antepartum vaginal and rectal swab cultures (7.4% by vaginal swab samples and 8.9% by rectal samples) (Table 1), and the PCR-strategy (intrapartum vaginal swab sample) found 12.2% GBS-positive women (Table 2).

**Table 1:** Concordance between detection of GBS colonization analyzed by antepartum culture (rectum and vaginal) and intrapartum culture (vagina) as the reference standard.

		Intrapartum vaginal culture (reference)		
		Positive	Negative	Total = 902
Antepartum culture		N (%)		
Vagina or rectum	Positive	85	71	156 (17.3)
	Negative	19	727	746
Vagina	Positive	67	33	100 (11.1)
	Negative	37	765	802
Rectum	Positive	80	66	146 (16.2)
	Negative	24	732	756

**Table 2:** Detection of GBS colonization by intrapartum polymerase chain reaction test (PCR) compared to intrapartum vaginal culture as the reference standard.

	Intrapartum vaginal culture (reference)		
	Positive	Negative	Total
Intrapartum vaginal PCR	N (%)		
	104 (11.5)	798 (88.5)	902 (100)
Positive	86 (82.7)	24	110 (12.2)
Negative	18	774 (98.1)	792 (87.8)

Based on the reference standard, the performance characteristics of the culture-strategy and the PCR-strategy are given in Tables 2 and 3. Notably, a marked difference between the positive likelihood ratios (LH+) of 9.2 for the culture-strategy and 27.5 for the PCR-strategy was seen. The positive predictive value was 55% for combining antepartum vaginal and rectal swab cultures and 78% for PCR-strategy.

**Table 3:** Performance of antepartum vaginal/rectal culture and intrapartum vaginal PCR test using intrapartum vaginal culture for GBS as the reference standard.

	Antepartum culture						Intrapartum PCR	
	For GBS						For GBS	
	Vagina		Rectum		Vagina or rectum		Vagina	
	%, n/N	95% CI	%, n/N	95% CI	%, n/N	95% CI	%, n/N	95% CI
Sensitivity	64 67/104	54–74	77 80/104	68–85	82 85/104	73–89	83 86/104	74–89
Specificity	96 765/798	94–97	92 732/798	90–94	91 727/798	89–93	97 774/798	96–98
PPV	67 67/100	55–74	55 80/146	46–63	55 85/156	46–63	78 86/110	69–86
NPV	95 765/802	94–97	97 732/756	95–98	98 727/746	96–99	98 774/792	96–99
LH+	16 65/1-96	11–22	9 77/1-92	7–12	9 82/1-91	7–12	27 83/1-97	18–41

CI = confidence interval; PPV = positive predictive value; NPV = negative predictive value; LH = Likelihood ratio

The false negative rate by the PCR-strategy was 17% (18/104). Fourteen of these 18 false negative samples were assessed by the semi-quantitative culture assessment, and among these 12 (86%) were classified with only few GBS colonies (Table 4). On the other hand, the false positive rate was 3% (24/798).

**Table 4:** Association between PCR and culture with semi-quantitative assessment of intrapartum vaginal growth of GBS in sample.

	Intrapartum vaginal culture (reference) (N = 902)				
	Positive				Negative
Intrapartum vaginal PCR	Semi-quantification				
	+	++	+++	Not assessed	
Positive	10	17	36	23	24
Negative	12	1	1	4	774

^+++^ = many

^++^ = some

^+^ = growth of few GBS colonies

## Discussion

We evaluated two screening strategies for identification of vaginal GBS colonization in a Danish cohort of laboring women, using an intrapartum culture as the reference standard. The antepartum culture-strategy achieved a LR+ of 9.2, whereas the intrapartum PCR-strategy achieved a LR+ of 27.5.

The strength of our study is the size of the cohort consisting of 902 participants from a well-defined population, which did not receive antibiotics between antepartum culture and the time of labor. It might be considered as a limitation that the PCR analyses were performed retrospectively as a batch processing of frozen samples thus only simulated as a rapid on-site test. However, to create a realistic screening scenario for a rapid PCR-strategy we used a GBS PCR assay without a delaying broth enrichment step prior to the PCR analysis. The Granada medium for isolation and identification of GBS is a selective and differential culture medium designed to selectively isolate *Streptococcus agalactiae* (Group B streptococcus, GBS) which differs from the standard recommended by CDC (Lim or TransVag).

The choice of a PCR assay for vaginal GBS detection performed without a prior Lim broth enrichment was intended and thereby also to accept a small, however, statistically significant lower sensitivity (92.7% versus 99.1%) compared to the use of the same PCR test with a Lim broth inoculation of the specimen according to the study of Silbert et al. [24]. Using a prior 18 hours Lim broth enrichment step as part of the PCR assay would prohibit the use of the GBS PCR as a rapid test at the time of delivery.

It may constitute a shortcoming of the study that omitting a prior enrichment step of the specimen is likely to reduce the number of positive cases detected by not only the PCR assay but also the intrapartum culture of the vaginal specimen. However, this approach allowed us to conduct the semi-quantitation of the GBS in the vaginal sample. These results indicate that the potential lower sensitivity of a PCR assay without a prior enrichment step with a false negative rate of 17% (18/104) is primarily caused by a failure to detect vaginal colonization with low numbers of GBS, which may be of less risk for the newborn during birth.

The false positive rate was only 3%, and in fact, we find it likely that these women may also be colonized with GBS, e.g., by non-hemolytic GBS isolates which may not be detectable on Granada agar plates. However, it is a limitation of the PCR-strategy that 3.4% of all specimens tested were initially undetermined for technical reasons based on the amplification status of the target and the Internal Process Control (data not shown). In such cases, a repeat testing must be conducted, which will delay the definitive result and may be in some cases not in time to decide the use of preventive antibiotic prophylaxis.

In contrast, antepartum screening by a GBS culture or PCR test with or without a prior Lim broth enrichment at week 35–37 during pregnancy is known to miss a substantial number of women with later intrapartum carriage of GBS [12, 13, 21, 27]. Furthermore, it should be noted that the difference in the detecting rates between the direct plating of the rectovaginal swab on the Granada medium and plating after prior Lim broth enrichment is only 4% [28].

Our study is the first of its kind performed in a country such as Denmark where the risk based approach is still recommended. This study is in line with prior studies reporting on the usefulness of a PCR-strategy in detecting intrapartum GBS [12, 14, 22, 29–31]. The GBS carriage rate was only 12% compared to 10–29% in other studies [27, 32–36] comprising different population and using other GBS detection methods based primarily on broth enrichment [2, 35, 37]. The difference between antepartum vaginal and rectal culture carriage (11% vs. 16%, respectively) have also been shown in previous studies, supporting the hypothesis that the gastrointestinal tract is the primary reservoir of GBS, and that vaginal colonization represents spread of GBS from rectum [34, 35].

Unlike other chromogenic media, the Granada medium cannot detect non-hemolytic GBS, thereby potentially decreasing the sensitivity of this culture medium for GBS screening [20]. However, the frequency of non-hemolytic GBS isolates is around 5% among GBS carriers, and a rate of only 1% is observed among invasive GBS strains, which suggests that EOGBS caused by non-hemolytic GBS strains is negligible [38].

The PCR strategy does not allow for performing antimicrobial susceptibility testing, which may be of relevance for penicillin-allergic patients. However, susceptibility testing is not necessary in general because GBS isolates with confirmed resistance to penicillin, ampicillin, or cefazolin have not yet been described [39]. Fortunately, efficient alternative choices exist for those with known penicillin-allergy, e.g., cefuroxime, cefaclor, and ceftriaxone. In patients with a history of severe anaphylactic reactions following cephalosporin treatment, vancomycin is an alternative antibiotic [40].

We conclude that in a Danish population of pregnant women with a low risk for their babies to acquire EOGBS, intrapartum PCR could be an efficient recommendation to screen for vaginal carriage of GBS during labor. It remains, to be evaluated, however from a medical technology perspective whether the test should be offered to all laboring women or only to those with a predefined risk. Such a medical technology evaluation must take into account 1) the fact that we have studied only proxy variables (GBS vaginal colonization) for EOGBS, 2) the overall costs, 3) the risks of maternal anaphylactic reactions and sensitization, 4) the possible adverse effects of antibiotics on the microbiome of the mother and the newborn [41], and 5) the risk of promoting drug resistance among the bacteria.

It should be noted that there are some practical demands of an intrapartum PCR test. It should first of all be simple for midwives or nurses to perform, and they should also be able to expect a test result made available within a relatively short time, which is necessary for the decision whether or not to administer antibiotics in a busy labor and delivery ward. In some urgent clinical cases, a PCR result may be required within less than 120 minutes, which is possible with the present PCR assay when a few patient samples are handled at a time.
